# Aflatoxin B_1_–Formamidopyrimidine DNA Adducts: Relationships between Structures, Free Energies, and Melting Temperatures

**DOI:** 10.3390/molecules24010150

**Published:** 2019-01-02

**Authors:** Martin Klvana, Urban Bren

**Affiliations:** 1 Laboratory of Physical Chemistry and Chemical Thermodynamics, Faculty of Chemistry and Chemical Technology, University of Maribor, Smetanova Ulica 17, 2000 Maribor, Slovenia; contact@martinklvana.com; 2 Laboratory for Molecular Modeling, Theory Department, National Institute of Chemistry, Hajdrihova 19, 1001 Ljubljana, Slovenia

**Keywords:** adduct, aflatoxin B_1_, DNA, formamidopyrimidine, linear interaction energy, linear response approximation, free energy, molecular dynamics simulation, molecular structure, thermal stability

## Abstract

Thermal stabilities of DNA duplexes containing Gua (*g*), α- (*a*) or β-anomer of formamidopyrimidine-N7-9-hydroxy-aflatoxin B_1_ (*b*) differ markedly (T_m_: a<g<b), but the underlying molecular origin of this experimentally observed phenomenon is yet to be identified and determined. Here, by employing explicit-solvent molecular dynamics simulations coupled with free-energy calculations using a combined linear-interaction-energy/linear-response-approximation approach, we explain the quantitative differences in T${}_{m}$ in terms of three structural features (bulkiness, order, and compactness) and three energetical contributions (non-polar, electrostatic, and preorganized-electrostatic), and thus advance the current understanding of the relationships between structures, free energies, and thermal stabilities of DNA double helices.

## 1. Introduction

**Figure 1:**
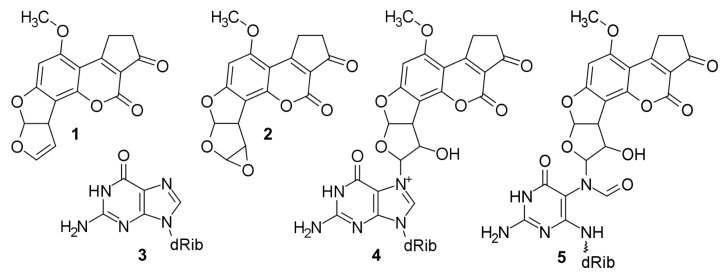
Structural formulae. **1**, AFB_1_; **2**, AFB1-E; **3**, Gua; **4**, Cationic Gua-N7-9-hydroxy-AFB_1_; **5**, FAPy-N7-9-hydroxy-AFB_1_.

Aflatoxin B_1_ (AFB_1_), systematically (6aR,9aS)-4-methoxy-2,3,6a,9a-tetrahydrocyclopenta[c]-[0]furo[3′,2′:4,5]furo[2,3-h]chromene-1,11-dione **1** (Figure 1) [1,2], a secondary metabolite [3] produced by aflatoxigenic [3] aspergilli [4,5,6,7,8,9,10] contaminates agricultural commodities (e.g., corn, peanuts, rice, sorghum, and wheat) [11] in tropical, subtropical, and temperate climate zones [12]. Following ingestion, inhalation, injection, or dermal absorption of matter contaminated with AFB_1_, and the subsequent cellular uptake of AFB_1_, the unsaturated lactone ring of AFB_1_ [13] is epoxidized [14,15,16,17]—in humans by various cytochrome P450 enzyme isoforms [18,19,20,21,22]—into metabolically activated [14,23,24,25,26,27] AFB_1_-8,9-epoxide **2** (AFB_1_-E; Figure 1) [15]. The exo isomer of AFB_1_-E [28,29,30,31] is an alkylating agent [13] that intercalates into both nuclear and mitochondrial dsDNA [31,32,33,34,35,36,37,38,39], and reacts with the N7 atom of Gua **3** (Figure 1) [36,40,41] to form, via bimolecular nucleophilic substitution [29,40], a covalent [16,17] cationic 8,9-dihydro-8-(N7-guanyl)-9-hydroxy-AFB_1_ adduct **4** (Figure 1) [40,42,43,44,45] whose AFB_1_ moiety is situated at the 5′-face of the modified Gua [34,35,39,46,47] and induces ∼20 degree bending of DNA [38]. **4** is thermally and alkali labile; therefore, **4** releases itself from DNA as 2,3-dihydro-2-(N7-guanyl)-9-hydroxy-AFB_1_ [40,46,48,49,50], leaving behind an abasic site [48], or transforms itself (by the opening of the imidazole ring of the modified Gua) into thermally stable 8,9-dihydro-8-(N5-formyl-2′,5′,6′-triamino-4-oxo-N5-pyrimidyl)-9-hydroxy-AFB_1_ **5** (FAPy-AFB_1_; Figure 1) [40,43,51,52], which remains firmly attached to the deoxyribose (dRib) in the native β-anomeric configuration (*b*) [39,53,54] and restores the original unbent DNA conformation [36,38].

The interconversion between *b* and the alternative α-anomer (*a*) occurs only in ssDNA [39,53,54], formed upon dissociation of the complementary strands of dsDNA during DNA replication, transcription, and repair. Although ssDNA shows a slight preference for *a*, re-association of the complementary DNA strands strongly favors *b* [39,53,54], which is a consequence of (1) *a* to *b* conversion in ssDNA being very slow, (2) ssDNA life-time being too brief, and (3) *b* being more conducive than *a* to the re-association [39]. *a* and *b* are strong blockers of DNA replication [13,50,53,55,56,57,58,59,60,61,62,63,64,65] and transcription [23,25,32,55,59,60,61,62,66,67,68,69]. Additionally, *b* is a strong mutagen for it is the material cause of the *b*·C→T·A transversion substitution mutation [46,49,53,64,70,71,72,73,74,75,76,77,78,79,80,81,82], the efficient cause of which is an erroneous bypass of *b* lesion by the translesion DNA polymerase ζ [64,65].

As a bulky adduct, *b* is a potential substrate for nucleotide excision repair (NER) [47,51,82,83,84,85,86,87,88], a complex mechanism involving more than 40 proteins and operating in two modes: global genome repair (GGR; sensitive to disrupted base-pairing at the site of the lesion) and transcription-coupled repair (TCR; sensitive to, and triggered by, the ability of the lesion to block the elongating RNA polymerase II complex) [86,88]. Owing to the intact Watson-Crick (WC) hydrogen-bonding interaction between β-FAPy and the complementary cytosine [36], *b* is only a very poor substrate for GGR [88], and thus the repair of *b* depends on TCR [81], which is restricted to the transcriptionally active regions of genomic DNA [89], and within these regions only to the transcribed strand [81]. Moreover, the efficiency of TCR is hampered by the binding of AFB_1_-induced lipid-peroxidation products (acetaldehyde and crotonaldehyde) to, and inhibiting the natural roles of, NER proteins [82]. Alternatively, **5** can be excised by the promiscuous DNA glycosylase NEIL1 [90], but the base excision repair pathway as a whole is inhibited by AFB_1_ [82].

The oxidative stress induced by AFB_1_ [82,91,92]; the conversion of AFB_1_ to AFB_1_-E; and the formation, stability, inefficient repair, and mutagentic potential of *b* and acetaladehyde (mHPG, α-methyl-γ-hydroxy-1,N2-propano-Gua) DNA adducts is the complex cause [82] of the cytotoxic [3,4,13,55,59,65,93,94,95,96,97,98] (hepatotoxic [93,94,95,97,99,100,101,102,103,104], nephrotoxic [102,105], pulmotoxic [56,106,107], immunotoxic [104,108], and neurotoxic [109]), terratogenic [110], tumorigenic, and carcinogenic effects associated with AFB_1_ [32,82,93,94,99,101,102,106,111,112,113,114,115] and modulated by cell- [97], tissue- [86], individual- [20,116], and species-specific susceptibility [15,55,110,117,118,119].

**Figure 2:**
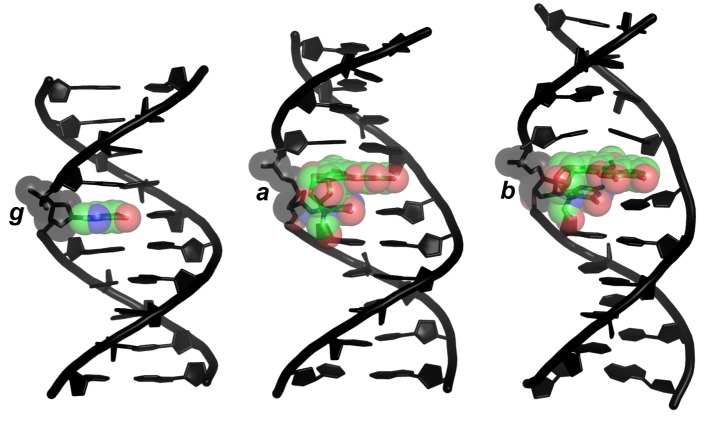
Structures of dsDNA decamers differing in the nucleobase 5. (***g***), Gua (standard B-DNA model); (***a***), α-FAPy-N7-9-hydroxy-AFB_1_ (NMR structure; PDB ID 2KH3); (***b***), β-FAPy-N7-9-hydroxy-AFB_1_ (NMR structure; PDB ID 1HM1). Green, carbon; blue, nitrogen; red, oxygen.

*b* can exert its harmful influence on DNA information content and retrieval if, and only if, it persists for a biologically meaningful time. In this regard, a peculiar characteristic of dsDNA oligonucleotides containing *b* (dsDNA*_b_*) is a higher, resistance-to-NER conferring [47], thermal stability (melting temperature, T_m_) of the duplex relative to dsDNA*_a_* (ΔT_m_ ∼ 27 K) and dsDNA*_g_* (ΔT_m_ ∼ 13 K) [33,34,36,38,39,47], which has been qualitatively (but not quantitatively) ascribed, based on nuclear magnetic resonance (NMR) structures of dsDNA*_a_* and dsDNA*_b_* (Figure 2), to favorable stacking interactions [27,36,38,39,47] (and not to perturbed WC hydrogen bonding interactions, for these remain intact in both dsDNA*_a_* and dsDNA*_b_*) [36,39,47].

And this brings us to defining the main object of our present inquiry, an attempt to answer the following question: *What are the structural and energetical causes, not only qualitatively but also quantitatively, of the experimentally observed differences in the thermal stability of dsDNA_g_, dsDNA_a_, and dsDNA_b_?* For knowing the answer to this question—besides being the good per se (as one of the pieces of the aflatoxin puzzle)—may help us to proceed from particular observations to general principles that determine the structure and stability of N5-substituted FAPy lesions and intercalated bulky DNA adducts on a long journey toward a complete understanding of the stability and energetics of the DNA double helix in terms of the contributions of polar and non-polar interactions [120].

We approach the problem of quantifying the structural and energetical causes of the differences in the thermal stability between dsDNA*_g_*, dsDNA*_a_*, and dsDNA*_b_* theoretically in three steps: (1) generating ensembles of structures-and-energies of dsDNA and ssDNA models of DNA*_g_* [121,122,123], DNA*_a_* [39], and DNA*_b_* [36] using molecular dynamics (MD) simulations [124], (2) calculating absolute (ΔG, dsDNA vs. ssDNA) and relative free energies (ΔΔG, *g* vs. *a* vs. *b*) using a combination [125] of linear response approximation (LRA) [126] and linear interaction energy (LIE) methods [127], and (3) correlating the experimentally known melting temperatures [39,47] with the ensemble-derived structural signatures and free energies.

## 2. Results

### 2.1. Sizes

*a* (α-FAPy-N7-9-hydroxy-AFB_1_) and *b* (β-FAPy-N7-9-hydroxy-AFB_1_) are of equal size and 3.3 times bulkier than *g* (Gua), from which the ranking by bulkiness was determined: g<a=b.

### 2.2. Structures

**Figure 3:**
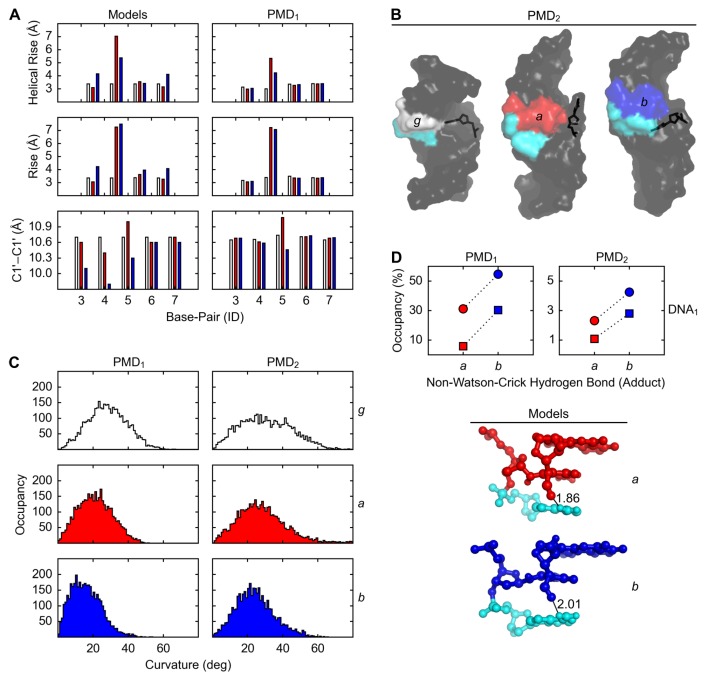
Geometrical features of DNA with Gua (*g*, white), α- (*a*, red) and β-FAPy-N7-9-hydroxy-AFB_1_ (*b*, blue) adducts. (**A**) Inter- and intra-base-pair parameters in DNA models and PMD_1_ simulations. (**B**) Perturbation of base-pair 5 in PMD_2_ simulations. (**C**) Angle between base-pair origins 1, 5, and 10 in PMD simulations. (**D**) Intra-strand hydrogen bond between nucleobases 5 (*a*, red; *b*, blue) and 6 (Ade, cyan) in PMD simulations and models of DNA_1_ (circles, dsDNA; squares, ssDNA; distances in Å).

The lengths of helical rise between the base pairs 4 and 5 in the initial dsDNA models vs. PMD_1_ (producing molecular dynamics; the polar state 1) structures of *g*, *a*, and *b* were, respectively, 3.4 vs. 3.0, 7.0 vs. 5.3, and 5.4 vs. 4.2 Å (the average of DNA_1_, the A·T variant, and DNA_2_, the G·C variant), from which the ranking by disorder was determined: g<b<a (Figure 3A, top row). In contrast to the length of helical rise, the length of rise between the base pairs 4 and 5 does not distinguish *a* from *b*: g<a=b (Figure 3A, middle row). The distances between the C1’ atoms of the base-pair 5 in the initial dsDNA models of *g*, *a*, and *b* were 10.7, 11.0, and 10.3 Å, respectively, and the average occupancies of WC-5 (the Watson-Crick hydrogen bonds within the base-pair 5) in PMD_2_ (the non-polar state 2) structures of *g*, *a*, and *b* were, respectively, 14, 11, and 17% (the average of DNA_1_ and DNA_2_); from these two measures the ranking by looseness was determined: b<g<a (Figure 3A, bottom row).

The same ranking, b<g<a, would be also obtained for the distances between the C1′ atoms of the base-pair 5 in PMD structures, but only if ST, significance threshold, for the differences were ignored, because the distances in PMD_1_ vs. PMD_2_ structures of *g*, *a*, and *b* were, respectively, 10.7 vs. 11.9, 11.1 vs. 12.3, and 10.5 vs. 11.8 Å (the average of DNA_1_ and DNA_2_), and so the differences (δ) between *g* and *b* were δ⩽ST=0.3 Å. The same ranking, b<g<a, would be also obtained for the occupancy of the perturbed conformation of the nucleobase Cyt-16 (Figure 3B), but only if ST for the differences in the occupancies were ignored, because the occupancies were 0.5, 1.6, and 0.2% (the average of DNA_1_ and DNA_2_), respectively, for *g*, *a*, and *b*, and so the differences (δ) between the occupancies were δ⩽ST=3%.

The dsDNA bending angles in PMD_1_ structures of *g*, *a*, and *b* were, respectively, 25, 20, and 15° (the average of DNA_1_ and DNA_2_), and the corresponding dsDNA bending angles in PMD_2_ structures were 30, 25, and 20°, respectively; from these two measures the ranking by curvature was determined: b<a<g (Figure 3C).

The average occupancies of nWC (the non-Watson-Crick hydrogen bond involving the formyl group of the FAPy moiety and the exocyclic amino group of the 3′-neighboring Ade in DNA_1_) in PMD_1_ structures of dsDNA*_a_*, dsDNA*_b_*, ssDNA*_a_*, and ssDNA*_b_* were 31, 55, 6, and 30%, respectively, and the corresponding occupancies in PMD_2_ structures were 2, 4, 1, and 3%, respectively; from these two measures the rankings by the stability of nWC in dsDNA_1_ and ssDNA_1_, and the ranking by percentual difference in the stability of nWC between dsDNA_1_ and ssDNA_1_, were determined: a<b, a<b, and a=b, respectively (Figure 3D).

### 2.3. Free Energies

Contributions of the probes to the absolute free energies of dsDNA formation (Table S1) were obtained from interaction energies (Table 1); relative free energies (Table 2) were obtained from the corresponding absolute free energies. ΔG_vdw1_ of *g*, *a*, and *b* were, respectively, −1.0, −2.9, and −3.0 kcal/mol (the average of DNA_1_ and DNA_2_), from which the ranking by non-electrostatic contribution of the probe to the dsDNA formation, was determined: a=b<g. ΔG_ele1_ of *g*, *a*, and *b* were, respectively, −2.7, −0.5, and −1.4 kcal/mol (the average of DNA_1_ and DNA_2_), from which the ranking by electrostatic contribution of the probe in the polar state 1 to the dsDNA formation, was determined: g<b<a. ΔG_ele2_ of *g*, *a*, and *b* were, respectively, −0.4, 0.7, and −1.2 kcal/mol (the average of DNA_1_ and DNA_2_), from which the ranking by electrostatic contribution of the probe in the non-polar state 2—i.e., the ranking by the contribution of electrostatic preorganization to the dsDNA formation—was determined: b<g<a. ΔG_ele_ of *g*, *a*, and *b* were, respectively, −3.1, 0.2, and −2.6 kcal/mol (the average of DNA_1_ and DNA_2_), from which the ranking by total electrostatic contribution of the probe to the dsDNA formation, was determined: g<b<a. ΔG of *g*, *a*, and *b* were, respectively, −4.1, −2.8, and −5.6 kcal/mol (the average of DNA_1_ and DNA_2_), from which the ranking by the total contribution of the probe to the free energy of the dsDNA formation, b<g<a, and the ranking by |ΔΔG|, using *g* as the reference, a=b, were determined. The differences in ΔG between DNA_2_ (the G·C variant) and DNA_1_ (the A·T variant), $\Delta \Delta G_{\mathrm{dna}}=\Delta G_{\mathrm{dna}2}-\Delta G_{\mathrm{dna}1}$, for *g*, *a*, and *b* were 0.4, −0.3, and 1.5 kcal/mol, respectively, from which the ratio of correct:incorrect signs of ΔΔG_dna_ was determined: 1:2.

The maximum average convergence errors of the scaled interaction energies, $\alpha E_{\mathrm{vdw}1}$, $\beta E_{\mathrm{ele}1}$, $\beta E_{\mathrm{ele}2}$, and $\beta E_{\mathrm{ele}}$ (obtained from PMD_1_ and PMD_2_ simulations of dsDNA*_g_*, dsDNA*_a_*, dsDNA*_b_*, ssDNA*_g_*, ssDNA*_a_*, and ssDNA*_b_*) were, respectively, 0.3, 0.9, 0.7, and 1.1 kcal/mol (the average of DNA_1_ and DNA_2_). The maximum average convergence errors of ΔG_vdw1_, ΔG_ele1_, ΔG_ele2_, ΔG_ele_, and ΔG were, respectively, 0.4, 1.5, 1.1, 2.0, and 2.2 kcal/mol (the average of DNA_1_ and DNA_2_). The maximum standard deviations of these free energies were, respectively, 0.3, 1.7, 1.2, 2.0, and 2.0 kcal/mol (the average of DNA_1_ and DNA_2_). The maximum spreads of these free energies were, respectively, 0.7, 3.9, 2.4, 4.8, and 4.4 kcal/mol (the average of DNA_1_ and DNA_2_). The ratios of low:medium:high uncertainties of these interaction and free energies were 3:1:0 and 1:3:1, respectively—as determined from the maximum average convergence errors using arbitrary, but judicious thresholds (kcal/mol): low⩽1.0<medium⩽2.0<high.

### 2.4. Correlations

**Figure 4:**
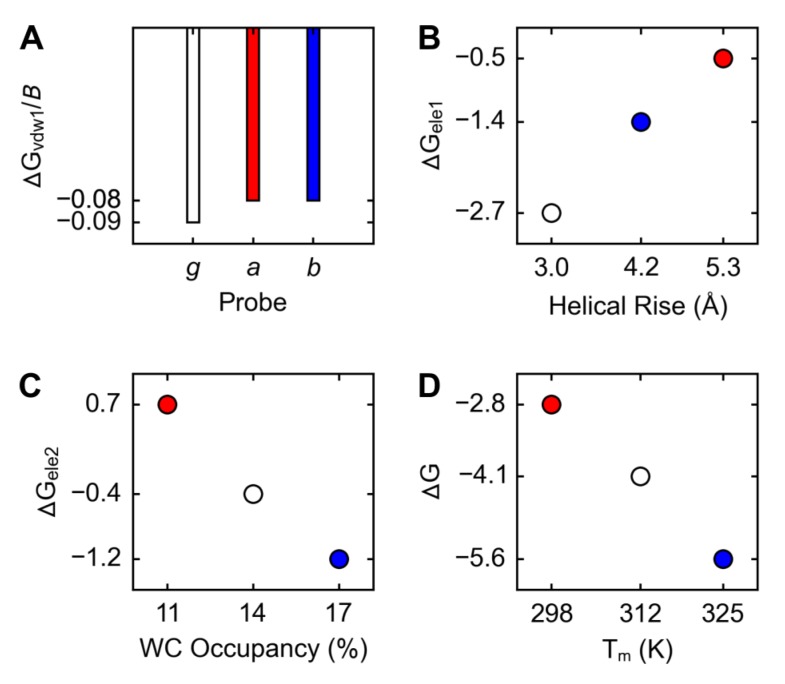
Relationships between molecular structures, free energies (kcal/mol), and melting temperatures of DNA containing Gua (*g*, white), α- (*a*, red) or β-FAPy-N7-9-hydroxy-AFB_1_ (*b*, blue) adduct. (**A**) Probe vs. average van der Waals free energy per non-hydrogen probe-atom. (**B**) Helical rise vs contribution of the probe to the electrostatic free energy of dsDNA formation obtained from PMD_1_ simulations. (**C**) Average occupancy of the Watson-Crick hydrogen bonds between nucleobases of the base pair 5 vs. contribution of the probe to the electrostatic free energy of dsDNA formation obtained from PMD_2_ simulations. (**D**) Melting temperature vs. contribution of the probe to the free energy of dsDNA formation.

The ranking by bulkiness, g<a=b, was the same as the inverse of the ranking by ΔG_vdw1_, a=b<g; hence, the bulkier the probe, the more favorable its non-electrostatic contribution to the free energy of dsDNA formation: $\Delta G_{\mathrm{vdw}1}\approx -0.1uB$, where *u* is 1 kcal/mol and *B* is the number of non-hydrogen atoms in the probe (Figure 4A). The ranking by disorder, g<b<a, was the same as the ranking by ΔG_ele1_; hence, the greater the disorder, the less favorable the electrostatic contribution from the polar state to the free energy of dsDNA formation: $\Delta \Delta G_{\mathrm{ele}1}\approx u\Delta D$, where *u* is 1 kcal/(mol·Å) and *D* is the helical rise obtained from PMD_1_ structures (Figure 4B). The ranking by looseness, b<g<a, was the same as the ranking by ΔG_ele2_ (Figure 4C); hence, the greater the looseness, the less favorable the electrostatic contribution from the non-polar state to the free energy of dsDNA formation: $\Delta \Delta G_{\mathrm{ele}2}\approx -0.3u\Delta L$, where *u* is 1 kcal/mol and *L* is the WC occupancy obtained from PMD_2_ structures. The ranking by ΔG, b<g<a, was the same as the inverse of the ranking by T_m_ (melting temperature); hence, the more favorable the free energy of dsDNA formation, the higher the melting temperature: $\Delta T_{m}\approx -10u\Delta \Delta G$, where *u* is 1 mol·K/kcal (Figure 4D).

## 3. Discussion

### 3.1. Relationships between Structures, Free Energies, and Melting Temperatures

Now, let us bring to the reader’s attention the main question to which we seek answers in our present, theoretical work (in which we simulate the molecular dynamics of dsDNA decamers and ssDNA trimers—containing *g*, *a*, or *b* as the probe of non-bonded interactions—in aqueous solution), *What are the structural and energetical causes, not only qualitatively but also quantitatively, of the experimentally observed differences in the thermal stability of dsDNA_g_, dsDNA_a_, and dsDNA_b_?*, and let us offer the reader an answer: The differences in the melting temperatures (a<g<b) can be explained (1) structurally by the differences in bulkiness (g<a=b; measured by the number of non-hydrogen atoms in the probe), disorder (g<b<a; measured by the average length of the helical rise between the base pairs 4 and 5 in the ensemble of PMD structures of dsDNA generated with the probe in the natural, charged state 1), and looseness (b<g<a; measured by the average occupancy of the WC hydrogen bonds between nucleobases belonging to the base pair 5 in the ensemble of PMD structures of dsDNA generated with the probe in the artificial, uncharged state 2), and (2) energetically by the differences in the non-electrostatic (a=b<g; ΔG_vdw1_, calculated from the Lennard-Jones van der Waals interaction energies), electrostatic (g<b<a; ΔG_ele1_, calculated from the Coulombic interaction energies obtained for the ensemble of PMD structures generated with the probe in its natural, charged state 1), and preorganized electrostatic (b<g<a; ΔG_ele2_, calculated from the Coulombic interaction energies obtained for the ensemble of PMD structures generated with the probe in its artificial, uncharged state 2) free energy contributions of a given probe to the total free energies of dsDNA formation (b<g<a; ΔG, calculated as the sum of ΔG_vdw1_, ΔG_ele1_, and ΔG_ele2_).

Thus, the three structural attributes, bulkiness (which anticorrelates linearly with ΔG_vdw1_), disorder (which correlates linearly with ΔG_ele1_), and looseness (which correlates linearly with ΔG_ele2_), determine ΔG (which correlates linearly with ΔG_ele2_ and anticorrelates linearly with the melting temperature, T_m_) as follows: the bulkier the nucleobase/adduct, and the less disordered the dsDNA, and the less loose the dsDNA, the higher the melting temperature. If the combined differences in the bulkiness (ΔG_vdw1_) and disorder (ΔG_ele1_) reflect the differences in the favorability of stacking interactions of *g*, *a*, and *b* with the nucleobases of the neighboring base-pairs—which, in general, is a reasonable assumption [128]—our results are qualitatively in agreement with other studies [36,38,39,47] but quantitatively unprecedented (for our computational work is the first of this kind). If the differences in ΔG_ele2_, quantified here for the first time, do indeed reflect the electrostatic preorganization of the WC hydrogen bonding interactions involving the nucleobases of the base pair 5, we have identified a hitherto unknown contribution to the differences in thermal stability, and the current view of the intactness of these interactions in both *a* and *b* adducts [36,39,47] might need to be reconsidered. However, compactness is the contrary to looseness, and what is compact, is put together closely, and thus the single best measure of looseness might be the distance between C1’ atoms of the base pair 5, which is the shortest in the NMR structure of dsDNA*_b_* [36] the longest in the NMR structure of dsDNA*_a_* [39] and intermediate in the standard B-DNA model of dsDNA*_g_* [121]. However, we did not find any explicit mentioning of these differences in the scientific literature. As for our PMD structures, they do, regardless of the charge state of the probe, preserve this ranking but only if we ignore our strict ST (significance threshold) of 0.3 Å. However, even if we do not ignore ST, which is our primary strategy, we can still clearly distinguish dsDNA*_a_* from dsDNA*_g_* from dsDNA*_b_* using this measure and so our current view is that the distance between C1’ atoms of the base pair 5 is indeed a useful indicator of looseness.

A peculiar feature of DNA_1_ is nWC (the intrastrand non-Watson-Crick hydrogen bond involving the formyl group of the FAPy moiety and the exocyclic amino group of the 3′-neighboring Ade) [36], which stabilizes the WC hydrogen-bonding interactions between the nucleobases in the base pairs 4–7 [36] but does not contribute to the differences in the melting temperature between DNA_1,*g*_, DNA_1,*a*_, and DNA_1,*b*_ [39,47], even though, compared to *a*, nWC involving *b* is more stable [39], and even though, compared to ssDNA, nWC in dsDNA is more stable [47]. In addition, while our PMD_1_ and PMD_2_ structures do not show the experimentally observed stabilizing effect of nWC on WC in the base pairs 4–7, they do agree with the remaining three experimental observations concerning nWC. Moreover, if our relative nWC occupancies (calculated as percentual differences) are quantitatively correct, the cause of the difference in the stability between nWC involving *a* and *b* resides in the geometric preferences of the adducts (and not in the differences between dsDNA and ssDNA).

### 3.2. Errors

Every measurement, no matter whether experimental or theoretical, is associated with errors: perfect measurement is impossible: every measurement is only approximate. In general, however, compared to experimental measurements, theoretical, computational results are prone to larger errors, because the latter do not actually observe real phenomena, but merely simulate (imitate) them, and they do so by using simplified models. The combined LRA-LIE approach, as employed in our present work, is no exception, despite the physical soundness and beautiful simplicity of the expression for the calculation of ΔG as the sum of ΔG_ele1_ (LRA), ΔG_ele2_ (LRA), and ΔG_vdw1_ (LIE) contributions [125,126,127,129,130]. Simply put, it is extremely difficult to calculate absolute binding free energies [127,129]; and the larger the molecules involved, the more degrees of freedom, and the bigger the problem [131]. Besides the problem with obtaining accurate ΔG values, there is also the issue of assessing the uncertainty of the ΔG values themselves [127]. We use three uncertainty metrics, namely, (1) convergence errors [127] (calculated as differences between ΔG values obtained from the first and second halves of PMD simulations), (2) standard deviations (calculated from ΔG values obtained from four parallel sets of PMD simulations), and (3) spreads (calculated as maximum differences between four parallel sets of ΔG values). While high convergence errors would imply that our 5.0 ns PMD simulations are too short, high standard deviations would imply that our four parallel sets of PMD simulations are too few; and large spreads would illustrate the necessity of generating multiple parallel sets of PMD simulations. The uncertainties in the rankings of the free energy values are low (ΔG_vdw1_: a<g and b<g; ΔG_ele1_: g<a; ΔG_ele2_: g<a), medium (ΔG_ele1_: g<b; ΔG: b<g), and high (ΔG_ele2_: b<g; ΔG: g<a), for the convergence errors smaller, similar (within ST of 0.3 kcal/mol), or greater than the unsigned free energies, and our confidence in the meaningfulness of the rankings are, correspondingly, high, medium, and low. However, no average convergence error in ΔG_vdw1_, ΔG_ele1_, ΔG_ele2_, and ΔG is greater than 0.4, 1.5, 1.1, and 2.2 kcal/mol, respectively, and, therefore, if we adopt 2.0 kcal/mol as the threshold of good convergence [127,130,132] only the convergence error of ΔG for DNA_*a*_, and only due to the convergence error of ΔG for DNA_2,*a*_, exceeds this good convergence threshold, and only by 0.2 kcal/mol. We would have to generate one or more additional sets of PMD simulations to lower the convergence error, but no improvement in the accuracy could be expected because our ΔG values for DNA*_g_*, DNA*_a_*, and DNA*_b_* are already perfectly linearly correlated with the corresponding melting temperatures.

### 3.3. Strengths and Weaknesses

Interpreting quantitative relationships based on three points requires extreme caution because the probability of fortuitous correlations is not negligible. We would like to emphasize that we use the original scaling parameters of the van der Waals (α=0.161) and electrostatic (β=0.500) interaction energies [127], because neither the refined scaling parameters (α=0.180, $\beta_{g}=0.430$, $\beta_{a}=\beta_{b}=0.370$) [133] nor free parametrization (all combinations of α and β from 0.000 to 1.000 by 0.001 increments) improves the linear correlation between the free energies and the corresponding melting temperatures, which is supportive of the physical meaning residing in the free energy contributions to ΔG, and we would caution against the use of α and β parameters as freely adjustable fudge factors if the purpose of obtaining binding free energies is truly scientific (Proclus): “For the task of science is the recognition of causes, and only when we recognize the causes of things do we say that we know them.” If the original α and β values do not result in a good agreement between the calculated free energies and the corresponding experimental quantities, it is, in our opinion, less likely, due to the lack of robustness of the combined LRA-LIE approach but, rather, due to a problem with the molecular model or due to an insufficient sampling of the configurational space. The latter is the probable reason why our ΔG rankings for DNA variants with swapped identities of the nucleobases in the base pair 6 with respect to DNA_1_ (T·A: g<b<a) and DNA_2_ (C·G: g=b<a) were incorrect (and therefore not included in the dataset used for the interpretation of the relationships between structures, free energies, and melting temperatures).

The reliability of this computational approach depends, to a certain extent, on the availability of the corresponding experimental quantities. In addition if the differences between the experimental quantities translate into sub-1.0 kcal/mol differences in the calculated free energies, such as, in our case of DNA_1_ vs. DNA_2_, it is difficult, if at all possible, to distinguish such small differences with high confidence, and the reason for this is, ultimately, as with any other computational or experimental technique, the detection limit, which is a function of both signal strength (sensitivity) and signal stability (noisiness). Only massively parallel PMD simulations would provide a definite answer about the true sensitivity and noisiness limits—and only for a given case, really, because the limits are partly case-specific—but such an undertaking is beyond the scope of our present work.

## 4. Materials and Methods

### 4.1. Structural Models

NMR structures of dsDNA decamers in B-conformation, PDB IDs 2KH3 [39] (*a*; model ID 1) and 1HM1 [36] (*b*; model ID 1) consisting of two complementary DNA strands (strand ID 1: 5′-CTATXYTTCA-3′, where X-5 is *a* or *b*, and Y-6 is Ade, A; and strand ID 2: 5′-TGAAZCATAG-3′, where Z-15 is Thy, T) were obtained from the Protein Data Bank [134], and named, for convenience, dsDNA_1,*a*_ and dsDNA_1,*b*_, respectively; dsDNA_2,*a*_ and dsDNA_2,*b*_ were generated from their corresponding dsDNA_1_ models by replacing the original nucleobases Y-6 and Z-15 with Gua and Cyt, respectively, for the purpose of exploring two sequence-specific effects: (1) an intra-strand non-WC hydrogen bond involving the formyl group of the FAPy moiety and the exocyclic amino group of the 3′-neighboring Ade, but not the Gua, and (2) two vs. three WC hydrogen bonds involving the complementary nucleobases Y·Z (A·T vs. G·C). Four single-stranded DNA models (ssDNA_1,*a*_, ssDNA_1,*b*_, ssDNA_2,*a*_, and ssDNA_2,*b*_), were created by extracting the nucleotides 4–6 from the corresponding dsDNA models (5′-TXY-3′, where X-2 is *a* or *b*, and Y-3 is Ade or Gua); and these trimeric models were considered to be suitable approximations of the corresponding decameric strands 1 in the dissociated, single-stranded configuration [135,136]. The reference dsDNA_1,*g*_ model in the standard B-conformation [121] was built using X3DNA 2.1 [122,123] (fiber −seq=CTATXYTTCA −b, where X-5 is Gua and Y-6 is Ade), and the three remaining models—dsDNA_2,*g*_, ssDNA_1,*g*_, and ssDNA_2,*g*_—were created analogously to the corresponding DNA*_a_* and DNA*_b_* models.

### 4.2. Energetical Models

Atom types, bond lengths, bond angles, torsion angles, and partial atomic charges of the natural nucleotides in the DNA models were described using the AMBER 95 Force Field [137,138]. The total charge of each 5′-terminal, non-terminal, and 3′-terminal natural nucleotide was, −0.3079, −1.0000, and −0.6921 *e*, respectively (amber11/data/leap/lib/DNA_CI.lib) [138]. Atom types, bond lengths, bond angles, and torsion angles of *a* and *b* were primarily described by the AMBER 95 Force Field [137], secondarily by the General AMBER Force Field (which is compatible with the AMBER 95 Force Field) [139], and tertiarily by the analogy to the AMBER 95 Force Field. Partial atomic charges of *a* and *b* were derived using the Restrained Electrostatic Potential (RESP) method [140], as implemented in AMBER 11 (resp −O −i resp.in −o resp.out −p resp.pch −t resp.chg −e esp) [138], applied to the quantum-mechanically calculated electrostatic potential (ESP) at the Hartree-Fock (HF) level of theory with 6-31G(d) basis set using Gaussian 09 (#HF 6−31G(d) opt scf = tight pop = MK iop(2/11 =1) iop(6/33 = 2)) [141] for 8-methyl-9-hydroxy-AFB_1_, with the carbon atom of the methyl group corresponding to the C1′ atom of the dRib to which the FAPy moiety is attached. The net charges of the methyl groups in 8-methyl-9-hydroxy-AFB_1_ were evenly distributed among all atoms of FAPy-N7-9-hydroxy-AFB_1_, and rounded to four decimal places; thus the original partial atomic charges of the dRib (including the atoms C1′ and H1′) and the net −1 *e* charge of X-5 nucleotide containing either *a* or *b* were preserved.

### 4.3. Solvation

The net −18 and −2 *e* charge of dsDNA and ssDNA models, respectively, was neutralized by an addition of 18 and 2 sodium ions (each with +1 *e* charge) on a grid surrounding the DNA: randomly, but not closer than 5 Å from any atom of the DNA, not farther than 18 Å from the geometrical center of the DNA, and not closer than 6 Å from each other. The resulting electroneutral complexes, composed of DNA and sodium ions, were immersed in a spherical grid—being centered at the geometrical center of DNA and having 28 Å in radius—of TIP3P water molecules [142] using the preparation program Qprep (version 5.03) from the molecular dynamics package Q (version 5.0) [124].

### 4.4. Simulation

The variable parts of the DNA*_g_*, DNA*_a_*, and DNA*_b_* models—i.e., *g* (15 atoms; net charge −0.0888 *e*), *a* (54 atoms; net charge −0.0888 *e*), and *b* (54 atoms; net charge −0.0888 *e*)—would be used as the probes (Tables S2 and S3; Figure S1), for which their van der Waals interaction energies (E_vdw_), modeled using Lennard-Jones potential, and their electrostatic interaction energies (E_ele_), modeled according to the Coulomb’s law, with all the components of the surrounding environment—i.e., with the probe-less part of the DNA, the ions, and all the water molecules—would be collected from MD simulations for these are the potential energies from which the van der Waals (ΔG_vdw_) and electrostatic (ΔG_ele_) contributions of the probe to the free energy of dsDNA formation (ΔG) would be obtained. MD simulations would be performed, separately, using the normally charged probe (state ID 1) and the uncharged probe (state ID 2; with all partial atomic charges set to 0 *e*), and the charged probe would be used for collecting E_vdw1_, E_ele1_, and E_ele2_, where the integers 1 and 2 denote the simulated state.

The solvated DNA models, containing the probe in the state 1, were prepared for the subsequent collecting of structures and energies by a well-tried, continuous series of 12 equilibrating MD simulations (EMD) [143] using the simulation program Qdyn (version 5.04) from the molecular dynamics simulation package Q (Table S4): Water molecules were subjected to the surface-constraint all-atom solvent (SCAAS)-type boundary conditions [124]. DNA was prevented from moving toward the boundary of the simulation sphere, but not hindered in its tumbling motion, by being restrained to its geometrical center with a force constant of 2.0 kcal/(mol·Å^2^) (dsDNA), or by having the C1′ atom of the nucleotide X-2 restrained to its initial coordinates with a force constant of 50 kcal/(mol·Å^2^) (ssDNA). No cut-off was applied to non-bonded interactions involving the atoms of the probe. Non-bonded interactions between atoms not belonging to the probe were evaluated explicitly and using the Local Reaction Field method for distances ⩽ and > 10 Å, respectively. Four parallel equilibrations were generated for each solvated DNA model by executing the equilibrating MD simulation protocol with four different values of the seed for the pseudo-random number generator (which is used by Qdyn to generate initial velocities). The collecting of structures (every 1.0 ps) and energies (every 0.02 ps) was performed in the last 5000 ps of 5000 ps (with the probe being in the state 1) and 5500 ps (with the probe being in the state 2) of 96 producing MD simulations (PMD_1_ and PMD_2_), each of which was a natural continuation of the 12th EMD simulation (ensemble, constant NVT; temperature, 298.15 K; step size, 2 fs; bonds involving hydrogen atoms restrained using the SHAKE algorithm). Hence, 5000/250,000 and 480,000/24,000,000 structural/energetical configurations were harvested per PMD simulation and in total, respectively.

### 4.5. Visualization

Molecular structures were visualized using PyMOL (version 0.99rc6) [144] and VMD (version 1.87) [145].

### 4.6. Measurement

The size of the probe was determined by the number of non-hydrogen atoms constituting the probe. Every 10th PMD structure was characterized—based on three attributes of bodies: locality, length, and angularity—using X3DNA. The presence of hydrogen-bonding interactions—both WC (involving complementary nucleobases, including FAPy) and non-WC (involving the formyl group of the FAPy moiety and the exocyclic amino group of the 3′-neighboring Ade)—was determined, for every PMD structure, using hydrogen ⋯ acceptor distance (⩽2.5 Å) and donor–hydrogen ⋯ acceptor angle (⩾135°) criteria, and hydrogen-bonding occupancies were calculated as fractions of the structures in the ensemble that satisfied these arbitrary, but stringent, geometrical standards for hydrogen-bonding [146]. The conformation of Cyt-16 in PMD structures of dsDNA was classified as perturbed when the distance between the geometrical centers of Cyt-16 and Gua- or FAPy-5 exceeded our arbitrary, but judicious, threshold of 8.0 Å.

The contribution of the probe to the absolute free energy of dsDNA formation was calculated from the average E_vdw1_, E_ele1_, and E_ele2_ interaction energies (〈⋯〉), which were extracted from the collections of energies from PMD simulations of dsDNA (ds) and ssDNA (ss) models using Qfep (version 5.01) from the molecular dynamics simulation package Q as follows (Equations (1)–(4)): [125]

(1) $$\Delta G=\Delta G_{\mathrm{vdw}1}+\Delta G_{\mathrm{ele}1}+\Delta G_{\mathrm{ele}2}$$

(2) $$\Delta G_{\mathrm{vdw}1}=\alpha \Delta E_{\mathrm{vdw}1}=\alpha \left(\langle E_{\mathrm{vdw}1,\mathrm{ds}}\rangle -\langle E_{\mathrm{vdw}1,\mathrm{ss}}\rangle \right)$$

(3) $$\Delta G_{\mathrm{ele}1}=\beta_{1}\Delta E_{\mathrm{ele}1}=\beta_{1}\left(\langle E_{\mathrm{ele}1,\mathrm{ds}}\rangle -\langle E_{\mathrm{ele}1,\mathrm{ss}}\rangle \right)$$

(4) $$\Delta G_{\mathrm{ele}2}=\beta_{2}\Delta E_{\mathrm{ele}2}=\beta_{2}\left(\langle E_{\mathrm{ele}2,\mathrm{ds}}\rangle -\langle E_{\mathrm{ele}2,\mathrm{ss}}\rangle \right)$$

where α=0.161 and $\beta_{1}=\beta_{2}=0.500=\beta$ [127,131]. The relative free energies of dsDNA_g_, dsDNA_a_, and dsDNA_b_ formation were calculated as differences between the corresponding absolute free energies (Equation (5)):

(5) $$\Delta \Delta G_{f-g}=\Delta G_{f}-\Delta G_{g}$$

where *f* is either *a* or *b*.

The structural and energetical quantities obtained from parallel PMD simulations were simply averaged. The rounding error was set, arbitrarily, but judiciously, to 0.1 Å for distances, 1.0° for angles, 1.0% for occupancies, and 0.1 kcal/mol for energies. The average energetical quantities were also calculated (1) separately for each of the four parallel sets of PMD simulations (for the purpose of assessing non-cumulative uncertainties in these quantities as standard deviations and spreads), and (2) separately for the first and last 130,000 energies (for the purpose of assessing non-cumulative convergences of these quantities as differences between the two averages). An arbitrary, but judicious, significance threshold (ST) of three times the rounding error was set for the structural and energetical differences between the DNA models distinguished by the identities of X-5, Y-6, and Z-15 nucleotides. The rankings of the DNA models according to the structural and energetical quantities, produced from the sums of integer significance scores obtained from the matrices of differences (δ) between the DNA models (−1, if δ⩽−ST; 0, if −ST<δ<ST; 1, if ST⩽δ), were compared with each other and with the ranking according to the experimental T_m_ (a<g<b). In the cases of matching rankings, linear correlation coefficients (R^2^; ST set to 0.96) were calculated by comparing the actual quantities, for the purpose of which only one set of experimental T_m_ values—representing, approximately (within the experimental error of 1 K), the average of DNA_1_ and DNA_2_—was used: $T_{m,g}=312$ K, $T_{m,a}=298$ K, and $T_{m,b}=325$ K, indicating that T_m,g_ lies, approximately, in the middle between T_m,*a*_ ($\Delta T_{m,a-g}=-14$ K) and T_m,*b*_ ($\Delta T_{m,b-g}=13$ K) [39,47].

## 5. Conclusions

Having identified the general attributes of *a* (bulky), *b* (as bulky as *a*), dsDNA*_a_* (disordered and loose) and dsDNA*_b_* (disordered, but less than dsDNA*_a_*, and compact), and thus having answered our main question, we ask ourselves: (1) How do the attributes of thermal (de)stabilization modulate (i) the efficiency and fidelity of the bypass of these lesions by the translesion-synthesis DNA polymerase ζ (which “preferentially misincorporates Ade opposite the lesion [64,65],” suggesting that this polymerase is “responsible for the predominant G·C→T·A mutation” induced by FAPy-AFB1 adducts) [64,65], (ii) the recognition of these lesions by the global-genome-repair-specific XPC-HR23B complex (which “screens the genome for damage on the basis of disrupted base-pairing instead of lesions per se”) [88], and (iii) the subsequent dual incision (which is the “rate-limiting step of the nucleotide excision repair [86],” and which releases an oligonucleotide containing the lesion) [147], and (2) why is the thermal stabilization of DNA duplex by *b* (which is the dominant FAPy-N7-9-hydroxy-AFB_1_ adduct in genomic DNA) [54] not common among bulky-and-intercalated-but-not-cross-linked DNA adducts [36]? These questions remain to be answered by future experimental and theoretical studies.

## Figures and Tables

**Table 1:** Scaled non-bonded interaction energies (kcal/mol). ^a^

	αE_vdw1_	βE_ele1_	βE_ele2_	βE_ele_
dsDNA_1g_	−4.4 (0.0, 0.0, 0.1)	−26.7 (0.4, 0.1, 0.2)	0.2 (0.1, 0.4, 0.8)	−26.5 (0.4, 0.4, 0.9)
dsDNA_1a_	−10.3 (0.1, 0.2, 0.4)	−50.6 (0.1, 2.1, 4.5)	−3.9 (0.6, 1.3, 3.1)	−54.4 (0.7, 2.5, 5.7)
dsDNA_1b_	−9.8 (0.0, 0.5, 1.0)	−50.6 (0.5, 2.5, 5.7)	−5.1 (0.6, 0.9, 1.9)	−55.8 (0.8, 3.0, 6.6)
ssDNA_1g_	−3.3 (0.2, 0.4, 0.8)	−24.3 (0.3, 0.5, 1.1)	0.9 (0.3, 0.1, 0.2)	−23.4 (0.4, 0.5, 1.1)
ssDNA_1a_	−6.8 (0.6, 0.4, 0.8)	−51.5 (1.1, 0.9, 2.1)	−3.8 (0.3, 0.4, 0.8)	−55.3 (1.0, 1.2, 2.7)
ssDNA_1b_	−7.1 (0.3, 0.3, 0.6)	−48.8 (0.9, 0.4, 0.8)	−3.4 (0.3, 0.2, 0.6)	−52.2 (1.2, 0.4, 0.9)
dsDNA_2g_	−4.4 (0.0, 0.0, 0.0)	−26.8 (0.3, 0.2, 0.6)	0.6 (1.3, 1.1, 2.7)	−26.1 (1.5, 1.1, 2.3)
dsDNA_2a_	−9.5 (0.1, 0.4, 0.7)	−51.8 (0.6, 1.6, 2.8)	−1.9 (1.3, 0.6, 1.3)	−53.7 (1.7, 1.7, 3.8)
dsDNA_2b_	−9.7 (0.2, 0.1, 0.3)	−50.2 (0.8, 0.8, 2.0)	−3.7 (0.7, 1.2, 2.5)	−53.9 (1.5, 1.3, 2.7)
ssDNA_2g_	−3.5 (0.3, 0.2, 0.4)	−23.8 (0.4, 0.2, 0.4)	0.7 (0.1, 0.2, 0.5)	−23.1 (0.4, 0.1, 0.1)
ssDNA_2a_	−7.2 (0.4, 0.1, 0.4)	−49.9 (0.7, 2.0, 4.8)	−3.3 (0.9, 0.6, 1.4)	−53.2 (1.5, 2.5, 5.9)
ssDNA_2b_	−6.5 (0.4, 0.4, 0.9)	−49.2 (0.9, 0.9, 2.0)	−3.1 (0.1, 0.2, 0.4)	−52.3 (0.8, 1.0, 2.3)
dsDNA_g_	−4.4 (0.0, 0.0, 0.0)	−26.7 (0.2, 0.1, 0.3)	0.4 (0.7, 0.5, 0.9)	−26.3 (0.6, 0.4, 0.9)
dsDNA_a_	−9.9 (0.1, 0.1, 0.2)	−51.2 (0.3, 1.8, 3.6)	−2.9 (0.6, 0.9, 1.9)	−54.1 (0.8, 2.0, 4.8)
dsDNA_b_	−9.8 (0.1, 0.2, 0.5)	−50.4 (0.7, 1.4, 3.2)	−4.4 (0.6, 0.9, 2.2)	−54.8 (1.1, 1.4, 3.4)
ssDNA_g_	−3.4 (0.2, 0.3, 0.6)	−24.0 (0.2, 0.3, 0.7)	0.8 (0.2, 0.1, 0.3)	−23.2 (0.4, 0.3, 0.5)
ssDNA_a_	−7.0 (0.3, 0.2, 0.5)	−50.7 (0.8, 0.8, 1.7)	−3.6 (0.5, 0.4, 0.8)	−54.2 (1.1, 1.1, 2.4)
ssDNA_b_	−6.8 (0.3, 0.3, 0.6)	−49.0 (0.8, 0.5, 1.2)	−3.2 (0.2, 0.1, 0.2)	−52.2 (0.9, 0.4, 1.1)

^a^α=0.161; β=0.500; vdw, van der Waals; ele, electrostatic; $E_{\mathrm{ele}}=E_{\mathrm{ele}1}+E_{\mathrm{ele}2}$; $\mathrm{DNA}=0.5({\mathrm{DNA}}_{1}+{\mathrm{DNA}}_{2})$; scaled interaction energy (convergence, standard deviation, spread).

**Table 2:** Relative free energies of dsDNA formation (kcal/mol). ^a^

	ΔΔG_vdw1_	ΔΔG_ele1_	ΔΔG_ele2_	ΔΔG_ele_	ΔΔG
DNA_1a_	−2.3 (0.6, 0.5, 1.3)	3.3 (0.9, 3.0, 6.8)	0.7 (0.3, 1.7, 4.2)	4.0 (1.1, 4.0, 8.7)	1.7 (1.3, 3.6, 7.7)
DNA_1b_	−1.6 (0.4, 0.6, 1.4)	0.6 (1.1, 2.5, 5.3)	−1.0 (0.9, 1.0, 2.2)	−0.4 (1.5, 2.8, 6.2)	−2.0 (1.6, 2.3, 5.3)
DNA_2a_	−1.5 (0.5, 0.6, 1.3)	1.0 (1.4, 1.2, 2.5)	1.6 (1.5, 2.3, 5.3)	2.6 (2.3, 3.0, 7.3)	1.1 (2.8, 3.2, 7.8)
DNA_2b_	−2.3 (0.6, 0.3, 0.7)	1.9 (1.8, 1.3, 3.1)	−0.5 (1.6, 2.2, 4.6)	1.4 (3.0, 2.8, 6.7)	−0.9 (2.5, 2.5, 6.0)
DNA_a_	−1.9 (0.4, 0.6, 1.3)	2.2 (1.1, 1.6, 3.7)	1.1 (0.7, 1.6, 3.1)	3.3 (1.6, 2.6, 6.2)	1.4 (1.9, 2.4, 5.2)
DNA_b_	−1.9 (0.5, 0.4, 0.9)	1.2 (1.4, 1.5, 3.6)	−0.7 (1.1, 1.3, 3.1)	0.5 (1.9, 1.7, 4.0)	−1.4 (1.4, 1.4, 3.1)

^a^ vdw, van der Waals; ele, electrostatic; $\Delta \Delta G_{\mathrm{ele}}=\Delta \Delta G_{\mathrm{ele}1}+\Delta \Delta G_{\mathrm{ele}2}$; $\Delta \Delta G=\Delta \Delta G_{\mathrm{vdw}1}+\Delta \Delta G_{\mathrm{ele}}$; $\mathrm{DNA}=0.5({\mathrm{DNA}}_{1}+{\mathrm{DNA}}_{2})$; reference systems: DNA_1g_, DNA_2g_, DNA_g_; free energy (convergence, standard deviation, spread).

## Supplementary Materials

The following are available online, Table S1: Contributions of the probes (*g*, *a*, or *b*) to the absolute free energy of dsDNA formation (kcal/mol), Table S2: Atom types and partial atomic charges of the Gua probe, Table S3: Atom types and partial atomic charges of the α- and β-FAPy-N7-9-hydroxy-AFB_1_ probes, Table S4: Parameters of 12-stage equilibrating molecular dynamics (EMD) simulations, Figure S1: Atom identifiers of the probes.

## Abbreviations

The following abbreviations are used in this manuscript:

AFB_1_	aflatoxin (*Aspergillus flavus* toxin) B_1_
*a*	α-FAPy-N7-9-hydroxy-AFB_1_
*b*	β-FAPy-N7-9-hydroxy-AFB_1_
*g*	Gua
AFB_1_-E	exo-8,9-epoxide of AFB_1_
dsDNA	double-stranded DNA
ΔΔG	relative free energy of dsDNA formation
ΔG	contribution of a probe (*g*, *a*, or *b*) to the absolute free energy of dsDNA formation
EMD	equilibrating MD simulation
FAPy	formamidopyrimidine
LIE	linear interaction energy
LRA	linear response approximation
MD	molecular dynamics
PMD	producing MD simulation
ssDNA	single-stranded DNA
T_m_	melting temperature
WC	Watson-Crick

## References

[1] Asao T., Buchi G., Abdel-Kader M.M., Chang S.B., Wick E.L., Wogan G.N. (1963). Aflatoxins B and G. J. Am. Chem. Soc..

[2] Asao T., Buechi G., Abdel-Kader M.M., Chang S.B., Wick E.L., Wogan G.N. (1965). The structures of aflatoxins B and G. J. Am. Chem. Soc..

[3] Nesbitt B.F., O’Kelly J., Sargeant K., Sheridan A. (1962). *Aspergillus flavus* and turkey X disease. Toxic metabolites of *Aspergillus flavus*. Nature.

[4] Sargeant K., Sheridan A., O’Kelly J., Carnaghan R.B.A. (1961). Toxicity associated with certain samples of groundnuts. Nature.

[5] Codner R.C., Sargeant K., Yeo R. (1963). Production of aflatoxin by the culture of strains of *Aspergillus flavus–oryzae* on sterilized peanuts. Biotechnol. Bioeng..

[6] Kurtzman C.P., Horn B.W., Hesseltine C.W. (1987). *Aspergillus nomius*, a new aflatoxin-producing species related to *Aspergillus flavus* and *Aspergillus tamarii*. Antonie van Leeuwenhoek.

[7] Klich M.A., Mullaney E.J., Daly C.B., Cary J.W. (2000). Molecular and physiological aspects of aflatoxin and sterigmatocystin biosynthesis by *Aspergillus tamarii* and *A. ochraceoroseus*. Appl. Microbiol. Biotechnol..

[8] Frisvad J.C., Skouboe P., Samson R.A. (2005). Taxonomic comparison of three different groups of aflatoxin producers and a new efficient producer of aflatoxin B_1_, sterigmatocystin and 3-*O*-methylsterigmatocystin, *Aspergillus rambellii* sp. nov. Syst. Appl. Microbiol..

[9] Varga J., Frisvad J.C., Samson R.A. (2011). Two new aflatoxin producing species, and an overview of *Aspergillus* section *Flavi*. Stud. Mycol..

[10] Carvajal-Campos A., Manizan A.L., Tadrist S., Akaki D.K., Koffi-Nevry R., Moore G.G., Fapohunda S.O., Bailly S., Montet D., Oswald I.P. (2017). *Aspergillus korhogoensis*, a novel aflatoxin producing species from the Côte d’Ivoire. Toxins.

[11] Hesseltine C.W., Shotwell O.L., Ellis J.J., Stubblefield R.D. (1966). Aflatoxin formation by *Aspergillus flavus*. Bacteriol. Rev..

[12] Battilani P., Toscano P., Van der Fels-Klerx H.J., Moretti A., Camardo Leggieri M., Brera C., Rortais A., Goumperis T., Robinson T. (2016). Aflatoxin B_1_ contamination in maize in Europe increases due to climate change. Sci. Rep..

[13] Legator M.S., Zuffante S.M., Harp A.R. (1965). Aflatoxin: Effect on cultured heteroploid human embryonic lung cells. Nature.

[14] Schoental R. (1970). Hepatotoxic activity of retrorsine, senkirkine and hydroxysenkirkine in newborn rats, and the role of epoxides in carcinogenesis by pyrrolizidine alkaloids and aflatoxins. Nature.

[15] Garner R.C. (1973). Chemical evidence for the formation of a reactive aflatoxin B_1_ metabolite, by hamster liver microsomes. FEBS Lett..

[16] Swenson D.H., Miller J.A., Miller E.C. (1973). 2,3-Dihydro-2,3-dihydroxy-aflatoxin B_1_: An acid hydrolysis product of an RNA–aflatoxin B_1_ adduct formed by hamster and rat liver microsomes in vitro. Biochem. Biophys. Res. Commun..

[17] Swenson D.H., Miller E.C., Miller J.A. (1974). Aflatoxin B_1_-2,3-oxide: Evidence for its formation in rat liver in vivo and by human liver microsomes in vitro. Biochem. Biophys. Res. Commun..

[18] Baertschi S.W., Raney K.D., Shimada T., Harris T.M., Guengerich F.P. (1989). Comparison of rates of enzymatic oxidation of aflatoxin B_1_, aflatoxin G_1_, and sterigmatocystin and activities of the epoxides in forming guanyl-N7 adducts and inducing different genetic responses. Chem. Res. Toxicol..

[19] Shimada T., Guengerich F.P. (1989). Evidence for cytochrome P-450_NF_, the nifedipine oxidase, being the principal enzyme involved in the bioactivation of aflatoxins in human liver. Proc. Natl. Acad. Sci. USA.

[20] Forrester L.M., Neal G.E., Judah D.J., Glancey M.J., Wolf C.R. (1990). Evidence for involvement of multiple forms of cytochrome P-450 in aflatoxin B_1_ metabolism in human liver. Proc. Natl. Acad. Sci. USA.

[21] Kamdem L.K., Meineke I., Gödtel-Armbrust U., Brockmöller J., Wojnowski L. (2006). Dominant contribution of P450 3A4 to the hepatic carcinogenic activation of aflatoxin B_1_. Chem. Res. Toxicol..

[22] He X.Y., Tang L., Wang S.L., Cai Q.S., Wang J.S., Hong J.Y. (2006). Efficient activation of aflatoxin B_1_ by cytochrome P450 2A13, an enzyme predominantly expressed in human respiratory tract. Int. J. Cancer.

[23] Roy A.K. (1968). Effects of aflatoxin B_1_ on polysomal profiles and RNA synthesis in rat liver. Biochim. Biophys. Acta.

[24] Goodall C.M., Butler W.H. (1969). Aflatoxin carcinogenesis: Inhibition of liver cancer induction in hypophysectomized rats. Int. J. Cancer.

[25] Edwards G.S., Wogan G.N. (1970). Aflatoxin inhibition of template activity of rat liver chromatin. Biochim. Biophys. Acta.

[26] Saunders F.C., Barker E.A., Smuckler E.A. (1972). Selective inhibition of nucleoplasmic rat liver DNA-dependent RNA polymerase by aflatoxin B_1_. Cancer Res..

[27] Ames B.N., Durston W.E., Yamasaki E., Lee F.D. (1973). Carcinogens are mutagens: A simple test system combining liver homogenates for activation and bacteria for detection. Proc. Natl. Acad. Sci. USA.

[28] Raney V.M., Harris T.M., Stone M.P. (1993). DNA conformation mediates aflatoxin B_1_-DNA binding and the formation of guanine N7 adducts by aflatoxin B_1_ 8,9-*exo*-epoxide. Chem. Res. Toxicol..

[29] Johnson W.W., Guengerich F.P. (1997). Reaction of aflatoxin B_1_
*exo*-8,9-epoxide with DNA: Kinetic analysis of covalent binding and DNA-induced hydrolysis. Proc. Natl. Acad. Sci. USA.

[30] Bren U., Guengerich F.P., Mavri J. (2007). Guanine alkylation by the potent carcinogen aflatoxin B_1_: Quantum chemical calculations. Chem. Res. Toxicol..

[31] Brown K.L., Bren U., Stone M.P., Guengerich F.P. (2009). Inherent stereospecificity in the reaction of aflatoxin B_1_ 8,9-epoxide with deoxyguanosine and efficiency of DNA catalysis. Chem. Res. Toxicol..

[32] Bhat N.K., Emeh J.K., Niranjan B.G., Avadhani N.G. (1982). Inhibition of mitochondrial protein synthesis during early stages of aflatoxin B_1_-induced hepatocarcinogenesis. Cancer Res..

[33] Gopalakrishnan S., Byrd S., Stone M.P., Harris T.M. (1989). Carcinogen-nucleic acid interactions: Equilibrium binding studies of aflatoxin B_1_ with the oligodeoxynucleotide d(ATGCAT)_2_ and with plasmid *pBR322* support intercalative association with the B-DNA helix. Biochemistry.

[34] Gopalakrishnan S., Harris T.M., Stone M.P. (1990). Intercalation of aflatoxin B_1_ in two oligodeoxynucleotide adducts: Comparative ^1^H NMR analysis of d(ATC^AFB^GAT)·d(ATCGAT) and d(AT^AFB^GCAT)_2_. Biochemistry.

[35] Johnston D.S., Stone M.P. (1995). Refined solution structure of 8,9-dihydro-8-(N7-guanyl)-9-hydroxyaflatoxin B_1_ opposite CpA in the complementary strand of an oligodeoxynucleotide duplex as determined by ^1^H NMR. Biochemistry.

[36] Mao H., Deng Z., Wang F., Harris T.M., Stone M.P. (1998). An intercalated and thermally stable FAPY adduct of aflatoxin B_1_ in a DNA duplex: Structural refinement from ^1^H NMR. Biochemistry.

[37] Nakatani K., Matsuno T., Adachi K., Hagihara S., Saito I. (2001). Selective intercalation of charge neutral intercalators into GG and CG steps: Implication of HOMO-LUMO interaction for sequence-selective drug intercalation into DNA. J. Am. Chem. Soc..

[38] Giri I., Stone M.P. (2002). Thermal stabilization of the DNA duplex by adducts of aflatoxin B_1_. Biopolymers.

[39] Brown K.L., Voehler M.W., Magee S.M., Harris C.M., Harris T.M., Stone M.P. (2009). Structural perturbations induced by the α-anomer of the aflatoxin B_1_ formamidopyrimidine adduct in duplex and single-strand DNA. J. Am. Chem. Soc..

[40] Croy R.G., Essigmann J.M., Reinhold V.N., Wogan G.N. (1978). Identification of the principal aflatoxin B_1_–DNA adduct formed in vivo in rat liver. Proc. Natl. Acad. Sci. USA.

[41] Lamm G., Pack G.R. (1990). Acidic domains around nucleic acids. Proc. Natl. Acad. Sci. USA.

[42] Essigmann J.M., Croy R.G., Nadzan A.M., Busby W.F., Reinhold V.N., Büchi G., Wogan G.N. (1977). Structural identification of the major DNA adduct formed by aflatoxin B_1_ in vitro. Proc. Natl. Acad. Sci. USA.

[43] Lin J.K., Miller J.A., Miller E.C. (1977). 2,3-Dihydro-2-(guan-7-yl)-3-hydroxy-aflatoxin B_1_, a major acid hydrolysis product of aflatoxin B_1_–DNA or –ribosomal RNA adducts formed in hepatic microsome-mediated reactions and in rat liver in vivo. Cancer Res..

[44] Martin C.N., Garner R.C. (1977). Aflatoxin B-oxide generated by chemical or enzymic oxidation of aflatoxin B_1_ causes guanine substitution in nucleic acids. Nature.

[45] Stark A.A., Essigmann J.M., Demain A.L., Skopek T.R., Wogan G.N. (1979). Aflatoxin B_1_ mutagenesis, DNA binding, and adduct formation in *Salmonella typhimurium*. Proc. Natl. Acad. Sci. USA.

[46] Bailey E.A., Iyer R.S., Stone M.P., Harris T.M., Essigmann J.M. (1996). Mutational properties of the primary aflatoxin B_1_–DNA adduct. Proc. Natl. Acad. Sci. USA.

[47] Li L., Brown K.L., Ma R., Stone M.P. (2015). DNA sequence modulates geometrical isomerism of the trans-8,9-dihydro-8-(2,6-diamino-4-oxo-3,4-dihydropyrimid-5-yl-formamido)-9-hydroxy aflatoxin B_1_ adduct. Chem. Res. Toxicol..

[48] Chu Y.H., Saffhill R. (1983). Errors in DNA synthesis induced by aflatoxin B_1_ modification of poly(dC-dG). Carcinogenesis.

[49] Foster P.L., Eisenstadt E., Miller J.H. (1983). Base substitution mutations induced by metabolically activated aflatoxin B_1_. Proc. Natl. Acad. Sci. USA.

[50] Refolo L.M., Conley M.P., Sambamurti K., Jacobsen J.S., Humayun M.Z. (1985). Sequence context effects in DNA replication blocks induced by aflatoxin B_1_. Proc. Natl. Acad. Sci. USA.

[51] Leadon S.A., Tyrrell R.M., Cerutti P.A. (1981). Excision repair of aflatoxin B_1_–DNA adducts in human fibroblasts. Cancer Res..

[52] Chetsanga C.J., Frenette G.P. (1983). Excision of aflatoxin B_1_–imidazole ring opened guanine adducts from DNA by formamidopyrimidine-DNA glycosylase. Carcinogenesis.

[53] Smela M.E., Hamm M.L., Henderson P.T., Harris C.M., Thomas H.M., Essigmann J.M. (2002). The aflatoxin B_1_ formamidopyrimidine adduct plays a major role in causing the types of mutations observed in human hepatocellular carcinoma. Proc. Natl. Acad. Sci. USA.

[54] Brown K.L., Deng J.Z., Iyer R.S., Iyer L.G., Voehler M.W., Stone M.P., Harris C.M., Harris T.M. (2006). Unraveling the aflatoxin–FAPY conundrum: Structural basis for differential replicative processing of isomeric forms of the formamidopyrimidine-type DNA adduct of aflatoxin B_1_. J. Am. Chem. Soc..

[55] Gabliks J., Schaeffer W., Friedman L., Wogan G. (1965). Effect of aflatoxin B_1_ on cell cultures. J. Bacteriol..

[56] Legator M. (1966). Biological effects of aflatoxin in cell culture. Bacteriol. Rev..

[57] Lillehoj E.B., Ciegler A. (1967). Inhibition of deoxyribonucleic acid synthesis in *Flavobacterium aurantiacum* by aflatoxin B_1_. J. Bacteriol..

[58] Wragg J.B., Ross V.C., Legator M.S. (1967). Effect of aflatoxin B_1_ on the deoxyribonucleic acid polymerase of *Escherichia coli*. Proc. Soc. Exp. Biol. Med..

[59] Harley E.H., Rees K.R., Cohen A. (1969). A comparative study of the effect of aflatoxin B_1_ and actinomycin D on HeLa cells. Biochem. J..

[60] Lafarge C., Frayssinet C. (1970). The reversibility of inhibition of RNA and DNA synthesis induced by aflatoxin in rat liver. A tentative explanation for carcinogenic mechanism. Int. J. Cancer.

[61] Maher V.M., Summers W.C. (1970). Mutagenic action of aflatoxin B_1_ on transforming DNA and inhibition of DNA template activity in vitro. Nature.

[62] Shieh J.C., Song P.S. (1980). Photochemically induced binding of aflatoxins to DNA and its effects on template activity. Cancer Res..

[63] Johnston D.S., Stone M.P. (2000). Replication of a site-specific trans-8,9-dihydro-8-(N7-guanyl)-9-hydroxyaflatoxin B_1_ adduct by the exonuclease deficient Klenow fragment of DNA polymerase I. Chem. Res. Toxicol..

[64] Lin Y.C., Li L., Makarova A.V., Burgers P.M., Stone M.P., Lloyd R.S. (2014). Molecular basis of aflatoxin-induced mutagenesis-role of the aflatoxin B_1_–formamidopyrimidine adduct. Carcinogenesis.

[65] Lin Y.C., Owen N., Minko I.G., Lange S.S., Li L., Stone M.P., Wood R.D., McCullough A.K., Lloyd R.S. (2016). DNA polymerase *ζ* limits chromosomal damage and promotes cell survival following aflatoxin exposure. Proc. Natl. Acad. Sci. USA.

[66] Gelboin H.V., Wortham J.S., Wilson R.G., Friedman M., Wogan G.N. (1966). Rapid and marked inhibition of rat-liver RNA polymerase by aflatoxin B_1_. Science.

[67] Sporn M.B., Dingman C.W., Phelps H.L., Wogan G.N. (1966). Aflatoxin B_1_: Binding to DNA in vitro and alteration of RNA metabolism in vivo. Science.

[68] Clifford J.I., Rees K.R. (1967). The action of aflatoxin B_1_ on the rat liver. Biochem. J..

[69] Neal G.E. (1972). The effect of aflatoxin B_1_ on normal and cortisol-stimulated rat liver ribonucleic acid synthesis. Biochem. J..

[70] Bressac B., Kew M., Wands J., Ozturk M. (1991). Selective G to T mutations of *p53* gene in hepatocellular carcinoma from southern Africa. Nature.

[71] Hsu I.C., Metcalf R.A., Sun T., Welsh J.A., Wang N.J., Harris C.C. (1991). Mutational hotspot in the *p53* gene in human hepatocellular carcinomas. Nature.

[72] Fujimoto Y., Hampton L.L., Luo L.D., Wirth P.J., Thorgeirsson S.S. (1992). Low frequency of *p53* gene mutation in tumors induced by aflatoxin B_1_ in nonhuman primates. Cancer Res..

[73] Aguilar F., Hussain S.P., Cerutti P. (1993). Aflatoxin B_1_ induces the transversion of G→T in codon 249 of the *p53* tumor suppressor gene in human hepatocytes. Proc. Natl. Acad. Sci. USA.

[74] Cariello N.F., Cui L., Skopek T.R. (1994). In vitro mutational spectrum of aflatoxin B_1_ in the human hypoxanthine guanine phosphoribosyltransferase gene. Cancer Res..

[75] Yang M., Zhou H., Kong R.Y., Fong W.F., Ren L.Q., Liao X.H., Wang Y., Zhuang W., Yang S. (1997). Mutations at codon 249 of *p53* gene in human hepatocellular carcinomas from Tongan, China. Mutat. Res..

[76] Courtemanche C., Anderson A. (1999). Multiple mutations in a shuttle vector modified by ultraviolet irradiation, (±)-7β,8α-dihydroxy-9α,10α-epoxy-7,8,9,10-tetrahydrobenzo[a]pyrene, and aflatoxin B_1_ have different properties than single mutations and may be generated during translesion synthesis. Mutat. Res..

[77] Denissenko M.F., Cahill J., Koudriakova T.B., Gerber N., Pfeifer G.P. (1999). Quantitation and mapping of aflatoxin B_1_-induced DNA damage in genomic DNA using aflatoxin B_1_-8,9-epoxide and microsomal activation systems. Mutat. Res..

[78] Pineau P., Marchio A., Battiston C., Cordina E., Russo A., Terris B., Qin L.X., Turlin B., Tang Z.Y., Mazzaferro V. (2008). Chromosome instability in human hepatocellular carcinoma depends on *p53* status and aflatoxin exposure. Mutat. Res..

[79] Paget V., Lechevrel M., Andre V., Goff J.L., Pottier D., Billet S., Garçon G., Shirali P., Sichel F. (2012). Benzo[a]pyrene, aflatoxin B_1_ and acetaldehyde mutational patterns in *TP53* gene using a functional assay: Relevance to human cancer aetiology. PLoS ONE.

[80] Chawanthayatham S., Valentine C.C., Fedeles B.I., Fox E.J., Loeb L.A., Levine S.S., Slocum S.L., Wogan G.N., Croy R.G., Essigmann J.M. (2017). Mutational spectra of aflatoxin B_1_ in vivo establish biomarkers of exposure for human hepatocellular carcinoma. Proc. Natl. Acad. Sci. USA.

[81] Letouzé E., Shinde J., Renault V., Couchy G., Blanc J.F., Tubacher E., Bayard Q., Bacq D., Meyer V., Semhoun J. (2017). Mutational signatures reveal the dynamic interplay of risk factors and cellular processes during liver tumorigenesis. Nat. Commun..

[82] Weng M.W., Lee H.W., Choi B., Wang H.T., Hu Y., Mehta M., Desai D., Amin S., Zheng Y., Tang M.S. (2017). AFB_1_ hepatocarcinogenesis is via lipid peroxidation that inhibits DNA repair, sensitizes mutation susceptibility and induces aldehyde–DNA adducts at *p53* mutational hotspot codon 249. Oncotarget.

[83] Sarasin A.R., Smith C.A., Hanawalt P.C. (1977). Repair of DNA in human cells after treatment with activated aflatoxin B_1_. Cancer Res..

[84] Oleykowski C.A., Mayernik J.A., Lim S.E., Groopman J.D., Grossman L., Wogan G.N., Yeung A.T. (1993). Repair of aflatoxin B_1_ DNA adducts by the UvrABC endonuclease of *Escherichia coli*. J. Biol. Chem..

[85] Alekseyev Y.O., Hamm M.L., Essigmann J.M. (2004). Aflatoxin B_1_ formamidopyrimidine adducts are preferentially repaired by the nucleotide excision repair pathway in vivo. Carcinogenesis.

[86] Bedard L.L., Alessi M., Davey S., Massey T.E. (2005). Susceptibility to aflatoxin B_1_-induced carcinogenesis correlates with tissue-specific differences in DNA repair activity in mouse and in rat. Cancer Res..

[87] Guo Y., Breeden L.L., Zarbl H., Preston B.D., Eaton D.L. (2005). Expression of a human cytochrome P450 in yeast permits analysis of pathways for response to and repair of aflatoxin-induced DNA damage. Mol. Cell. Biol..

[88] Bedard L.L., Massey T.E. (2006). Aflatoxin B_1_-induced DNA damage and its repair. Cancer Lett..

[89] Irvin T.R., Wogan G.N. (1984). Quantitation of aflatoxin B_1_ adduction within the ribosomal RNA gene sequences of rat liver DNA. Proc. Natl. Acad. Sci. USA.

[90] Vartanian V., Minko I.G., Chawanthayatham S., Egner P.A., Lin Y.C., Earley L.F., Makar R., Eng J.R., Camp M.T., Li L. (2017). NEIL1 protects against aflatoxin-induced hepatocellular carcinoma in mice. Proc. Natl. Acad. Sci. USA.

[91] Shen H.M., Shi C.Y., Lee H.P., Ong C.N. (1994). Aflatoxin B_1_-induced lipid peroxidation in rat liver. Toxicol. Appl. Pharmacol..

[92] Shen H.M., Ong C.N., Lee B.L., Shi C.Y. (1995). Aflatoxin B_1_-induced 8-hydroxydeoxyguanosine formation in rat hepatic DNA. Carcinogenesis.

[93] Lancaster M.C., Jenkins F.P., Philp J.M. (1961). Toxicity associated with certain samples of groundnuts. Nature.

[94] Butler W.H., Barnes J.M. (1963). Toxic effects of groundnut meal containing aflatoxin to rats and guinea-pigs. Br. J. Cancer.

[95] Carnaghan R.B.A., Hartley R.D., O’Kelly J. (1963). Toxicity and fluorescence properties of the aflatoxins. Nature.

[96] Tulpule P.G., Madhavan T.V., Gopalan C. (1964). Effect of feeding aflatoxin to young monkeys. Lancet.

[97] Judah D.J., Legg R.F., Neal G.E. (1977). Development of resistance to cytotoxicity during aflatoxin carcinogenesis. Nature.

[98] Paini A., Scholz G., Marin-Kuan M., Schilter B., O’Brien J., van Bladeren P.J., Rietjens I.M.C.M. (2011). Quantitative comparison between in vivo DNA adduct formation from exposure to selected DNA-reactive carcinogens, natural background levels of DNA adduct formation and tumour incidence in rodent bioassays. Mutagenesis.

[99] Barnes J., Butler W.H. (1964). Carcinogenic activity of aflatoxin to rats. Nature.

[100] Butler W.H. (1964). Acute toxicity of aflatoxin B_1_ in rats. Br. J. Cancer.

[101] Carnaghan R.B. (1967). Hepatic tumours and other chronic liver changes in rats following a single oral administration of aflatoxin. Br. J. Cancer.

[102] Epstein S.M., Bartus B., Farber E. (1969). Renal epithelial neoplasms induced in male Wistar rats by oral aflatoxin B_1_. Cancer Res..

[103] Alpert M.E., Hutt M.S., Wogan G.N., Davidson C.S. (1971). Association between aflatoxin content of food and hepatoma frequency in Uganda. Cancer.

[104] Lutwick L.I. (1979). Relation between aflatoxin, hepatitis-B virus, and hepatocellular carcinoma. Lancet.

[105] Grosman M.E., Elías M.M., Comin E.J., Rodriguez Garay E.A. (1983). Alterations in renal function induced by aflatoxin B_1_ in the rat. Toxicol. Appl. Pharmacol..

[106] Wieder R., Wogan G.N., Shimkin M.B. (1968). Pulmonary tumors in strain A mice given injections of aflatoxin B_1_. J. Natl. Cancer Inst..

[107] Paget V., Sichel F., Garon D., Lechevrel M. (2008). Aflatoxin B_1_-induced *TP53* mutational pattern in normal human cells using the FASAY (Functional Analysis of Separated Alleles in Yeast). Mutat. Res..

[108] Pier A.C., Heddleston K.L. (1970). The effect of aflatoxin on immunity in turkeys. I. Impairment of actively acquired resistance to bacterial challenge. Avian Dis..

[109] Ikegwuonu F.I. (1983). The neurotoxicity of aflatoxin B_1_ in the rat. Toxicology.

[110] DiPaolo J.A., Elis J., Erwin H. (1967). Teratogenic response by hamsters, rats and mice to aflatoxin B_1_. Nature.

[111] Dickens F., Jones H.E. (1963). The carcinogenic action of aflatoxin after its subcutaneous injection in the rat. Br. J. Cancer.

[112] Carnaghan R.B.A. (1965). Hepatic tumours in ducks fed a low level of toxic groundnut meal. Nature.

[113] Callen D.F., Mohn G.R., Ong T.M. (1977). Comparison of the genetic activity of aflatoxins B_1_ and G_1_ in *Escherichia coli* and *Saccharomyces cerevisiae*. Mutat. Res..

[114] Aguilar F., Harris C.C., Sun T., Hollstein M., Cerutti P. (1994). Geographic variation of *p53* mutational profile in nonmalignant human liver. Science.

[115] Liu Y., Wu F. (2010). Global burden of aflatoxin-induced hepatocellular carcinoma: A risk assessment. Environ. Health Perspect..

[116] McGlynn K.A., Rosvold E.A., Lustbader E.D., Hu Y., Clapper M.L., Zhou T., Wild C.P., Xia X.L., Baffoe-Bonnie A., Ofori-Adjei D. (1995). Susceptibility to hepatocellular carcinoma is associated with genetic variation in the enzymatic detoxification of aflatoxin B_1_. Proc. Natl. Acad. Sci. USA.

[117] Lancaster M.C. (1968). Comparative aspects of aflatoxin-induced hepatic tumors. Cancer Res..

[118] Portman R.S., Plowman K.M., Campbell T.C. (1968). Aflatoxin metabolism by liver microsomal preparations of two different species. Biochem. Biophys. Res. Commun..

[119] Newberne P.M., Butler W.H. (1969). Acute and chronic effects of aflatoxin on the liver of domestic and laboratory animals: A review. Cancer Res..

[120] Vologodskii A., Frank-Kamenetskii M.D. (2018). DNA melting and energetics of the double helix. Phys. Life Rev..

[121] Chandrasekaran R., Arnott S. (1996). The structure of B-DNA in oriented fibers. J. Biomol. Struct. Dyn..

[122] Lu X.J., Olson W.K. (2003). 3DNA: A software package for the analysis, rebuilding and visualization of three-dimensional nucleic acid structures. Nucl. Acids Res..

[123] Lu X.J., Olson W.K. (2008). 3DNA: A versatile, integrated software system for the analysis, rebuilding and visualization of three-dimensional nucleic-acid structures. Nat. Protoc..

[124] Marelius J., Kolmodin K., Feierberg I., Åqvist J. (1998). Q: A molecular dynamics program for free energy calculations and empirical valence bond simulations in biomolecular systems. J. Mol. Graph. Model..

[125] Ishikita H., Warshel A. (2008). Predicting drug-resistant mutations of HIV protease. Angew. Chem. Int. Ed. Engl..

[126] Lee F.S., Chu Z.T., Bolger M.B., Warshel A. (1992). Calculations of antibody-antigen interactions: Microscopic and semi-microscopic evaluation of the free energies of binding of phosphorylcholine analogs to McPC603. Protein Eng..

[127] Åqvist J., Medina C., Samuelsson J.E. (1994). A new method for predicting binding affinity in computer-aided drug design. Protein Eng..

[128] Šponer J., Šponer J.E., Mládek A., Jurečka P., Banáš P., Otyepka M. (2013). Nature and magnitude of aromatic base stacking in DNA and RNA: quantum chemistry, molecular mechanics, and experiment. Biopolymers.

[129] Singh N., Warshel A. (2010). Absolute binding free energy calculations: On the accuracy of computational scoring of protein-ligand interactions. Proteins.

[130] Gutiérrez-de Terán H., Åqvist J. (2012). Linear interaction energy: Method and applications in drug design. Methods Mol. Biol..

[131] Sham Y.Y., Chu Z.T., Tao H., Warshel A. (2000). Examining methods for calculations of binding free energies: LRA, LIE, PDLD-LRA, and PDLD/S-LRA calculations of ligands binding to an HIV protease. Proteins.

[132] Díaz L., Bujons J., Delgado A., Gutiérrez-de Terán H., Åqvist J. (2011). Computational prediction of structure-activity relationships for the binding of aminocyclitols to β-glucocerebrosidase. J. Chem. Inf. Model..

[133] Hansson T., Marelius J., Åqvist J. (1998). Ligand binding affinity prediction by linear interaction energy methods. J. Comput. Aided Mol. Des..

[134] Berman H.M., Westbrook J., Feng Z., Gilliland G., Bhat T.N., Weissig H., Shindyalov I.N., Bourne P.E. (2000). The Protein Data Bank. Nucl. Acids Res..

[135] Florián J., Goodman M.F., Warshel A. (2000). Free-energy perturbation calculations of DNA destabilization by base substitutions: The effect of neutral guanine·thymine, adenine·cytosine and adenine·difluorotoluene mismatches. J. Phys. Chem. B.

[136] Bren U., Lah J., Bren M., Martínek V., Florián J. (2010). DNA duplex stability: The role of preorganized electrostatics. J. Phys. Chem. B.

[137] Cornell W.D., Cieplak P., Bayly C.I., Gould I.R., Merz K.M., Ferguson D.M., Spellmeyer D.C., Fox T., Caldwell J.W., Kollman P.A. (1995). A second generation force field for the simulation of proteins, nucleic acids, and organic molecules. J. Am. Chem. Soc..

[138] Case D.A., Darden T.A., Cheatham T.E., Simmerling C.L., Wang J., Duke R.E., Luo R., Walker R.C., Zhang W., Merz K.M. (2010). AMBER.

[139] Wang J., Wolf R.M., Caldwell J.W., Kollman P.A., Case D.A. (2004). Development and testing of a general Amber force field. J. Comput. Chem..

[140] Bayly C.I., Cieplak P., Cornell W., Kollman P.A. (1993). A well-behaved electrostatic potential based method using charge restraints for deriving atomic charges: The RESP model. J. Phys. Chem..

[141] Frisch M.J., Trucks G.W., Schlegel H.B., Scuseria G.E., Robb M.A., Cheeseman J.R., Scalmani G., Barone V., Mennucci B., Petersson G.A. (2009). Gaussian.

[142] Jorgensen W.L., Chandrasekhar J., Madura J.D., Impey R.W., Klein M.L. (1983). Comparison of simple potential functions for simulating liquid water. J. Chem. Phys..

[143] Klvaňa M., Bren U., Florián J. (2016). Uniform free-energy profiles of the P–O Bond formation and cleavage reactions catalyzed by DNA polymerases β and λ. J. Phys. Chem. B.

[144] DeLano W.L. (2006). The PyMOL Molecular Graphics System.

[145] Humphrey W., Dalke A., Schulten K. (1996). VMD: Visual molecular dynamics. J. Mol. Graph..

[146] Arunan E., Desiraju G.R., Klein R.A., Sadlej J., Scheiner S., Alkorta I., Clary D.C., Crabtree R.H., Dannenberg J.J., Hobza P. (2011). Definition of the hydrogen bond (IUPAC Recommendations 2011). Pure Appl. Chem..

[147] Svoboda D.L., Taylor J.S., Hearst J.E., Sancar A. (1993). DNA repair by eukaryotic nucleotide excision nuclease. Removal of thymine dimer and psoralen monoadduct by HeLa cell-free extract and of thymine dimer by *Xenopus laevis* oocytes. J. Biol. Chem..

