# Supplementary material for: Aflatoxin B1–Formamidopyrimidine DNA Adducts: Relationships between Structures, Free Energies, and Melting Temperatures
Source: Molecules. 2019 Jan 2;24(1):150. doi: 10.3390/molecules24010150 (PMC6337653; doi:10.3390/molecules24010150)
Supplement: Supplementary file 1 [file molecules-24-00150-s001.zip › molecules-411474-SI.pdf]

Supplementary Materials: Aflatoxin B_1–Formamido-pyrimidine DNA Adducts: Relationships between Structures, Free Energies, and Melting Temperatures

Martin Klvana and Urban Bren

Table S1. Contributions of the probes (*g*, *a*, or *b*) to the absolute free energy of dsDNA formation (kcal/mol).[a]

	ΔG_{vdw1}	ΔG_{ele1}	ΔG_{ele2}	ΔG_{ele}	ΔG
DNA_{1g}	−1.2 (0.2, 0.4, 0.8)	−2.4 (0.5, 0.4, 0.9)	−0.7 (0.3, 0.3, 0.6)	−3.1 (0.7, 0.6, 1.5)	−4.3 (0.7, 0.4, 0.8)
DNA_{1a}	−3.5 (0.5, 0.2, 0.5)	0.9 (1.1, 2.6, 6.0)	−0.0 (0.6, 1.5, 3.7)	0.9 (1.2, 3.6, 8.1)	−2.6 (1.4, 3.5, 7.9)
DNA_{1b}	−2.8 (0.3, 0.3, 0.7)	−1.8 (1.4, 2.4, 5.6)	−1.7 (0.8, 1.0, 2.1)	−3.6 (2.0, 2.8, 5.7)	−6.3 (1.9, 2.5, 5.2)
DNA_{2g}	−0.9 (0.3, 0.2, 0.5)	−2.9 (0.7, 0.4, 0.9)	−0.1 (1.3, 1.1, 2.6)	−3.1 (1.5, 1.0, 2.3)	−3.9 (1.4, 1.0, 2.4)
DNA_{2a}	−2.4 (0.4, 0.4, 0.9)	−1.9 (1.0, 1.3, 2.4)	1.4 (2.2, 1.2, 2.7)	−0.5 (3.2, 2.1, 5.1)	−2.9 (3.6, 2.4, 5.4)
DNA_{2b}	−3.2 (0.6, 0.5, 1.2)	−1.0 (1.5, 1.7, 4.0)	−0.6 (0.8, 1.2, 2.6)	−1.6 (2.0, 1.9, 4.4)	−4.8 (1.6, 1.5, 3.6)
DNA_{g}	−1.0 (0.2, 0.3, 0.6)	−2.7 (0.4, 0.4, 0.9)	−0.4 (0.8, 0.5, 1.0)	−3.1 (0.9, 0.6, 1.4)	−4.1 (0.8, 0.4, 0.8)
DNA_{a}	−2.9 (0.3, 0.3, 0.7)	−0.5 (1.1, 1.3, 3.1)	0.7 (1.1, 1.2, 2.3)	0.2 (2.0, 2.0, 4.8)	−2.8 (2.2, 2.0, 4.4)
DNA_{b}	−3.0 (0.4, 0.3, 0.7)	−1.4 (1.5, 1.7, 3.9)	−1.2 (0.7, 1.0, 2.4)	−2.6 (2.0, 1.5, 3.4)	−5.6 (1.8, 1.3, 3.0)

[a]vdw, van der Waals; ele, electrostatic; $\Delta G_{vdw1} = \Delta G_{vdw1,dsDNA} - \Delta G_{vdw1,ssDNA}$; $\Delta G_{ele1} = \Delta G_{ele1,dsDNA} - \Delta G_{ele1,ssDNA}$; $\Delta G_{ele2} = \Delta G_{ele2,dsDNA} - \Delta G_{ele2,ssDNA}$; $\Delta G_{ele} = \Delta G_{ele1} + \Delta G_{ele2}$; $\Delta G = \Delta G_{vdw1} + \Delta G_{ele}$; DNA $= 0.5(DNA_1 + DNA_2)$; free energy (convergence, standard deviation, spread).

Table S2. Atom types and partial atomic charges of the Gua probe.[a]

ID	name	type	charge	ID	name	type	charge
13	N9	Nstar	0.0577	21	H1	H	0.3520
14	C8	CK	0.0736	22	C2	CA	0.7432
15	H8	H5	0.1997	23	N2	N2	−0.9230
16	N7	NB	−0.5725	24	H21	H	0.4235
17	C5	CB	0.1991	25	H22	H	0.4235
18	C6	C	0.4918	26	N3	NC	−0.6636
19	O6	O	−0.5699	27	C4	CB	0.1814
20	N1	NA	−0.5053				

[a]See Figure S1 for the assignment of atom identifiers.

Table S3. Atom types and partial atomic charges of the α- and β-FAPy-N7-9-hydroxy-AFB$_1$ probes.[a]

ID	name	type	charge	ID	name	type	charge
13	N13	N2	−0.3058	40	O40	OS	−0.2550
14	H14	H	0.2923	41	C41	C	0.6236
15	C15	CM	0.2157	42	O42	O	−0.5040
16	N16	NC	−0.5696	43	C43	CB	−0.1562
17	C17	CA	0.6487	44	C44	C	0.5077
18	N18	N2	−0.9042	45	O45	O	−0.5126
19	H19	H	0.4068	46	C46	CT	−0.2010
20	H20	H	0 .4068	47	H47	HC	0.0883
21	N21	NA	−0.3703	48	H48	HC	0.0883
22	H22	H	0.3250	49	C49	CT	−0.0333
23	C23	C	0.3519	50	H50	HC	0.0587
24	O24	O	−0.5406	51	H51	HC	0.0587
25	C25	CM	0.1326	52	C52	CB	0.0349
26	N26	N	−0.0720	53	C53	CA	−0.0326
27	C27	C	0.3594	54	C54	CA	0.0493
28	H28	H5	0.0662	55	O55	OS	−0.1767
29	O29	O	−0.5229	56	C56	CT	−0.0784
30	C30	CT	0.0468	57	H57	H1	0.0878
31	H31	H2	0.1424	58	H58	H1	0.0878
32	C32	CT	0.1831	59	H59	H1	0.0878
33	H33	H1	0.0786	60	C60	CA	−0.1003
34	O34	OH	−0.6592	61	H61	HA	0.1486
35	H35	HO	0.4221	62	C62	CB	0.0546
36	C36	CT	0.0502	63	O63	OS	−0.3414
37	H37	HC	0.0426	64	C64	CT	0.4616
38	C38	CB	−0.1040	65	H65	H2	0.0488
39	C39	CA	0.1299	66	O66	OS	−0.4363

[a]See Figure S1 for the assignment of atom identifiers.

Table S4. Parameters of 12-stage equilibrating molecular dynamics (EMD) simulations.

parameter	1	2	3	4	5	6	7	8	9	10	11	12
MD												
steps	10000	10000	10000	10000	20000	20000	20000	10000	10000	10000	20000	50000
stepsize	0.01	0.04	0.1	0.3	0.5	0.5	0.5	1.0	1.0	1.0	1.0	2.0
initial temperature	5.0											
temperature	5.0	5.0	5.0	5.0	5.0	5.0	50.0	150.0	200.0	250.0	298.0	298.0
bath coupling	0.1	0.4	1.0	3.0	40.0	40.0	40.0	40.0	40.0	40.0	40.0	40.0
random seed	S[a]											
shake solute	off	off	off	off	off	off	off	off	off	off	off	off
shake solvent	off	off	off	off	off	off	off	off	off	off	on	on
shake hydrogens	off	off	off	off	off	off	off	off	off	off	on	on
lrf	on	on	on	on	on	on	on	on	on	on	on	on
cut-offs												
solute solute	10.0	10.0	10.0	10.0	10.0	10.0	10.0	10.0	10.0	10.0	10.0	10.0
solvent-solvent	10.0	10.0	10.0	10.0	10.0	10.0	10.0	10.0	10.0	10.0	10.0	10.0
solute-solvent	10.0	10.0	10.0	10.0	10.0	10.0	10.0	10.0	10.0	10.0	10.0	10.0
q-atom	99.0	99.0	99.0	99.0	99.0	99.0	99.0	99.0	99.0	99.0	99.0	99.0
lrf	99.0	99.0	99.0	99.0	99.0	99.0	99.0	99.0	99.0	99.0	99.0	99.0
sphere												
shell force	10.0	10.0	10.0	10.0	10.0	10.0	10.0	10.0	10.0	10.0	10.0	10.0
shell radius	21.0	21.0	21.0	21.0	21.0	21.0	21.0	21.0	21.0	21.0	21.0	21.0
exclude bonded	off	off	off	off	off	off	off	off	off	off	off	off
solvent												
polarisation	on	on	on	on	on	on	on	on	on	on	on	on
charge correction	on	on	on	on	on	on	on	on	on	on	on	on
polarisation force	20.0	20.0	20.0	20.0	20.0	20.0	20.0	20.0	20.0	20.0	20.0	20.0
intervals												
non bond	10	10	10	10	10	10	10	10	10	10	10	10
output	500	500	500	500	500	500	500	500	500	500	500	500
temperature	10	10	10	10	10	10	10	10	10	10	10	10
energy	0	0	0	0	0	0	0	0	0	0	0	0
trajectory	500	500	500	500	500	500	500	500	500	500	500	500
volume change	0	0	0	0	0	0	0	0	0	0	0	0

[a]S: 1111 (EMD set ID 1), 2222 (EMD set ID 2), 3333 (EMD set ID 3), or 4444 (EMD set ID 4).

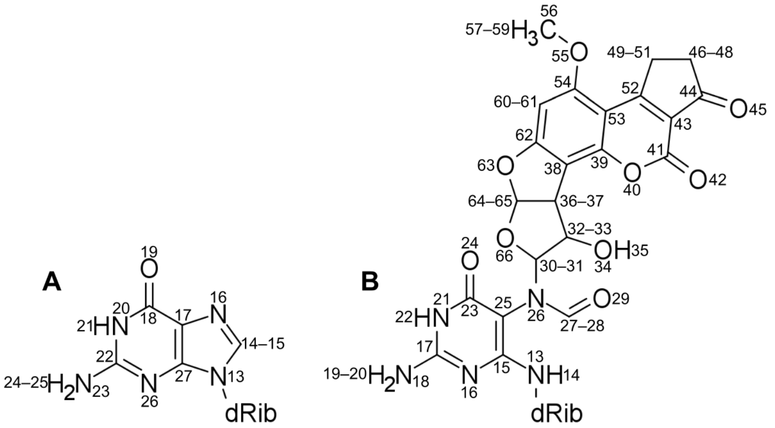

Figure S1. Atom identifiers of the probes: (**A**) Gua. (**B**) FAPy-N7-9-hydroxy-AFB$_1$.
